# Significant reduction of ischemia‐reperfusion cell death in mouse myocardial infarcts using the immediate‐acting PrC‐210 ROS‐scavenger

**DOI:** 10.1002/prp2.500

**Published:** 2019-07-12

**Authors:** Timothy A. Hacker, Gaoussou Diarra, Bryan L. Fahl, Susan Back, Erin Kaufmann, William E. Fahl

**Affiliations:** ^1^ Cardiovascular Physiology Core Facility, Department of Medicine University of Wisconsin‐Madison Madison Wisconsin; ^2^ Wisconsin Institutes for Medical Research University of Wisconsin‐Madison Madison Wisconsin

**Keywords:** hydrogen peroxide, neonate cardiomyocyte, troponin

## Abstract

Managing myocardial infarction (MI) to reduce cardiac cell death relies primarily on timely reperfusion of the affected coronary site, but reperfusion itself induces cell death through a toxic, ROS‐mediated process. In this study, we determined whether the PrC‐210 aminothiol ROS‐scavenger could prevent ROS‐induced damage in post‐MI hearts. In a series of both in vitro and in vivo experiments, we show that: (a) in vitro, PrC‐210 was the most potent and effective ROS‐scavenger when functionally compared to eight of the most commonly studied antioxidants in the MI literature, (b) in vitro PrC‐210 ROS‐scavenging efficacy was both immediate (seconds) and long‐lasting (hours), which would make it effective in both (1)* real‐time *(*seconds*), as post‐MI or cardiac surgery hearts are reperfused with PrC‐210‐containing blood, and (2)* long‐term *(*hour*s)*,* as hearts are bathed with systemic PrC‐210 after MI or surgery, (c) systemic PrC‐210 caused a significant 36% reduction of mouse cardiac muscle death following a 45‐minute cardiac IR insult; in a striking coincidence, the PrC‐210 36% reduction in cardiac muscle death equals the 36% of the MI‐induced cardiac cell death estimated 6 years ago by Ovize and colleagues to result from “reperfusion injury,” (d) hearts in PrC‐210‐treated mice performed better than controls after heart attacks when functionally analyzed using echocardiography, and (e) the PrC‐210 ROS‐scavenging mechanism of action was corroborated by its ability to prevent >85% of the direct, H_2_O_2_‐induced killing of neonate cardiomyocytes in cell culture. PrC‐210 does not cause the nausea, emesis, nor hypotension that preclude clinical use of the WR‐1065/amifostine aminothiol. PrC‐210 is a highly effective ROS‐scavenger that significantly reduces IR injury‐associated cardiac cell death.

**Abbreviations**
MI: myocardial infarction
TTC: triphenyltetrazolium chloride

## 1 INTRODUCTION

It is essential to re‐establish blood flow (reperfusion) to the heart after blockage (ischemia) in a coronary artery. While reperfusion will ultimately reduce the overall size of the myocardial infarct,^20^ the reperfused oxygenated blood, itself, significantly contributes to cardiac cell death.^14^ Restoration of blood flow after ischemia results in the *en masse* formation of highly toxic reactive oxygen species (ROS) in myocardial cells,^32^ which causes cardiomyocyte death and long‐term reduction in myocardial function.^30^.

Bigger et al^1^ reported that the prognosis for ischemic heart disease patients is inversely related to the extent of postmyocardial infarct tissue necrosis. The total annual cost of cardiovascular disease in the US is estimated at over $315 billion accounting for 15% of total health expenditures, more than any other disease.^12^ At $315 billion/year, any strategy that conferred a significant reduction in MI severity and tissue loss would have a significant impact to reduce health care costs, thus finding new ways to prevent MI‐associated cardiac necrosis remains an important goal.

Animal and clinical research demonstrate that oxygen free radicals cause tissue damage during myocardial ischemia‐reperfusion.^16^, ^18^ The bolus of oxygen that accompanies heart reperfusion generates ROS that simply exceed the cardiomyocyte detoxifying capability and reach levels that cause cardiomyocyte death. Davies et al^6^ showed that the levels of several species of reduced oxygen are increased in myocardial tissue during postischemic reperfusion of the heart, both *acutely* (seconds) during reperfusion, and *longer term,* that is, hours/days, from inflammatory cell‐generated ROS at the post‐MI site.^24^

PrC‐210 is a new direct‐acting, actually *immediate‐acting,* aminothiol ROS‐scavenger^5^, ^22^ that can be administered IV, orally, or topically, and it has no measurable nausea/emesis nor hypotension side effects.^26^ PrC‐210 is unlike virtually all of the previously tested “antioxidants” in the cardiac ischemia‐reperfusion literature. Earlier studied antioxidants either: (a) like Vitamin E, act *indirectly* over hours‐days via NrF‐2 to activate expression of protective genes,^28^ (b) like IV‐administered 50 kDa enzymes such as superoxide‐dismutase or catalase, detoxify ROS but cannot impact millisecond half‐life ROS generated inside reperfused cardiomyocytes, or (c) like adenosine or allopurinol, can possibly affect long‐term oxygen metabolism in mammalian cells, but have no impact upon ROS generated in reperfused cardiomyocytes.

Rather, PrC‐210 *directly* scavenges ROS; it has conferred 100% protection in seconds‐minutes against ROS‐induced cell kill^2^, ^5^ in previous radioprotection and ROS‐insult experiments.

In this study, we first determined PrC‐210 efficacy and potency to prevent an •OH insult, head‐to‐head in vitro against eight other “antioxidants” commonly studied in the cardiac ischemia‐reperfusion literature. Because PrC‐210 performed the best, we asked whether it could prevent ROS‐induced cell death in mouse hearts using the standard mouse heart ischemia‐reperfusion model. Finally, to corroborate the PrC‐210 mechanism of action, we directly exposed primary neonate mouse cardiomyocytes to an H_2_O_2_ insult and directly measured the PrC‐210 time‐ and dose‐dependency to prevent the H_2_O_2_‐induced cardiomyocyte death.

## 2 MATERIALS AND METHODS

### 2.1 Materials

Tissue culture media was from Gibco (ThermoFisher), fetal bovine serum was from Hyclone (Logan, UT), and culture‐ware was from Falcon. Thirty percent H_2_O_2_ was from Mallinckrodt. Synthesis of the PrC‐210 HCl aminothiol is described separately.^4^, ^9^ PrC‐210 HCl crystals are stored under a nitrogen atmosphere at −20°C, and even with routine thawing, use, and re‐storage, crystalline PrC‐210 is completely stable for >4 years by mass spectrometry analysis. Other chemical reagents were obtained from Sigma Aldrich (St. Louis, MO). C57 mice were from Jackson Laboratory (Bar Harbor, ME). Mice were maintained on 12‐hour light/dark cycle and provided ad lib water and food. All animal procedures were conducted according to a protocol (#M05714) approved by the University of Wisconsin Institutional Animal Care and Use Committee.

### 2.2 Mouse myocardial infarction (MI) model and PrC‐210 treatment

Adult male C57 mice (8‐10 weeks of age, 18‐22 g) were divided into two MI treatment groups^25^, ^27^: intraperitoneal (IP) vehicle or IP PrC‐210. At −20 minutes (ie, 20 minutes before coronary artery ligation), mice received an IP injection of either saline or PrC‐210 in water (252 µg/gm bw; pH 6.0). At −10 minutes, mice were initially anesthetized with 4% isoflurane and then maintained with 1%‐1.5% isoflurane during surgery. Mice were intubated and ventilated using a Minivent ventilator (Harvard Apparatus, March‐Hugstetten, Germany) at 200 breaths/minute. A left thoracotomy was performed, and at 0 minute the left coronary artery was ligated immediately after it emerged past the tip of the left atria using 6.0 Ethilon (Johnson and Johnson, Somerville, New Jersey) suture passed with a tapered needle. At +35‐40 minutes, mice received a second IP injection of either saline or PrC‐210 (101 µg/gm bw). At +45 minutes, the left coronary ligation was released and removed, the lungs were re‐expanded and the chest was closed. The animals were removed from the ventilator and allowed to recover on a heating pad.

### 2.3 Myocardial infarct size

The myocardial infarct size was evaluated 24 hours after the 45‐minute coronary artery ligation using Evan's Blue‐triphenyltetrazolium chloride (TTC) double staining. Following the 24‐hour reperfusion, 200  µL of a 2% solution of Evan's Blue dye was injected into the right jugular vein of the mouse. Five minutes after the injection, mouse hearts were rapidly removed, washed with a phosphate buffer solution and frozen at −20°C for at least 30 minutes. The entire heart was then cut into about five cross‐sectional slices, and the sections were then incubated in 1% TTC at 37°C for 15 minutes; sections were then fixed in 10% formalin solution. The infarct area (white color) and area at risk (red and white color) were digitally imaged in each heart slice, and white, red, and blue areas in each slice were traced. JPG images of every heart section were analyzed using Image J software and areas (ie, Total slice area, white, red, and blue areas of each slice) were then summed and calculations of White Area/Total Red + White Area for each heart were determined. This gives the MI size as a percentage of the area at risk.

### 2.4 Mouse heart echocardiography

In both control and PrC‐210‐treated mice, transthoracic echocardiography was conducted at 14 days and 28 days following 45‐minute infarct to assess left ventricular morphology and function in vivo. As previously described, mice were anesthetized with 5% isoflurane and then maintained with 1%‐2% isoflurane and room air throughout the procedure; body temperature was maintained at 37°C using a heated platform.^11^, ^13^ Echocardiographic parameters were measured over at least three consecutive cardiac cycles and averaged.

### 2.5 Mouse blood collection and troponin I ELISA

Twenty‐four hours after release of the coronary artery ligation, 100‐200 µL of blood was collected from mice via the jugular vein under isoflurane anesthesia; the blood was spun briefly in a 0.5 ml Eppendorf tube, and the supernatant was stored at −80°C prior to Troponin I assay. Troponin I levels were measured in mouse plasma and mouse cardiomyocyte tissue culture media using the Mouse Cardiac Troponin I ELISA Kit (Fisher #NC9402211). Absorbance (450 nm) in 96 wells was measured using a CLARIOStar plate reader.

### 2.6 Neonate mouse primary cardiomyocyte cultures

Primary cultures of neonatal mouse cardiomyocytes from 3 to 4‐day‐old C57 mice were prepared and cultured as described,^29^ with minor modification. Minced hearts were digested for 45 minutes with 0.2 mg/mL collagenase I (Sigma # C0130) at 37°C, the digest was poured through a 40 µm sterile sieve, cells were pelleted, and resuspended in RPMI plus 10% FBS and penicillin/streptomycin. The dissociated cells were plated in a standard 100 mm dish (Falcon) at 37°C for 1 hour to enrich the supernate in nonadherent cardiomyocytes. The supernate cardiomyocytes were collected and counted with a hemocytometer, and cells in fresh RPMI/10% FBS were then plated at 15‐100 × 10^3^ cells per well of a 96 well, Matrigel‐coated plate (Corning BioCoat Matrigel Matrix, Fisher Scientific).

The next day, medium was removed by aspiration with a 25 g needle, replaced with serum‐free RPMI, and PrC‐210 dilutions and/or H_2_O_2_ dilutions were added directly to the wells. After 2 hours at 37°C, the medium in wells was aspirated and replaced by RPMI plus 10% FBS and penicillin‐streptomycin. Twenty‐four hours later, 20 µL of CellTiter reagent (Promega, Madison, WI) was added to each well, and after 3 hours at 37°C, fluorescence of the bioconverted CellTiter reagent was read in a ClarioStar plate reader (excitation: 560 nm; emission: 590 nm). For Troponin I analysis, 50 µL of the RPMI media in wells at the end of the 2 hours H_2_O_2_ and PrC‐210 treatments was transferred to a new 96 well plate; the plate was sealed with Parafilm, and stored at −80°C until the Troponin I ELISA assay was done.

### 2.7 ROS‐induced naked DNA damage assay

**Figure 1:**
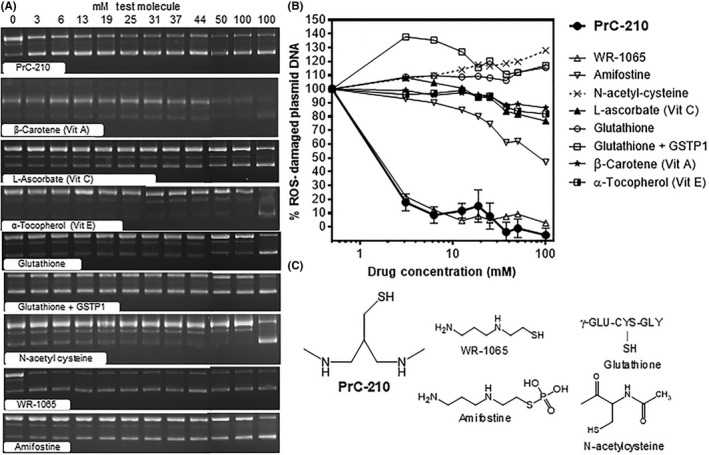
Small molecule protection of naked plasmid DNA from ROS‐induced damage. Fifteen minutes after test molecules were added to incubations, tubes were exposed to ROS insult. (A) Aliquots of incubations were electrophoresed and quantitative imaging of ethidium bromide stained gels was done. Intensities of lower (supercoiled) and upper (nicked/ROS‐damaged) bands were quantified using Image J software. (B) Mean band intensities were plotted using Graphpad Prism software. (C) Structures of some of the small molecules are shown

The dose‐dependent ability of the tested “antioxidants” to scavenge •OH was measured by their ability to prevent •OH‐induced breaks in a plasmid DNA molecule. pGEM plasmid DNA was purified from overnight cultures of E coli. For ROS‐protection assays, DNA (915 ng) was added to 40 µL incubations in PCR tubes containing 30 mmol L^−1^ sodium acetate buffer (pH 7.0). To assess ROS‐scavenger efficacy, 10 ul of each test molecule, at the same 12 increasing concentrations (0‐100 mmol L^−1^ final), were added to triplicate, plasmid‐containing tubes (assay TV = 50 µL). If test molecule was not sufficiently water soluble (eg, β‐carotene) Triton X‐100 was added to facilitate dispersion. Following a 15‐minute incubation at room temperature, the 36 experimental tubes and controls were exposed to an ROS insult by irradiation in an XRAD 320 irradiator. Aliquots of each irradiated tube were then mixed with gel loading dye and electrophoresed in a 1% agarose gel at 60 volts for 90 minutes. Gels were stained with ethidium bromide, quantitatively imaged, and Image J software was used to quantify supercoiled and nicked band intensity (both pre‐ and postirradiation) in each of the 36 sample lanes. Plots of “% ROS‐Damaged Plasmid DNA” vs protective drug concentrations enabled direct comparison of the protective efficacy of each tested molecule. All of the molecules tested in Figure 1 were obtained from Sigma‐Aldrich, except PrC‐210 which was synthesized as described above.

### 2.8 PrC‐210 protective lead time assay

To establish the lead time needed for PrC‐210 to protect naked DNA against a short‐lived •OH pulse, analogous to the •OH insult encountered in a postischemic reperfused heart, pGEM DNA (750 ng), serving as a surrogate •OH target, was incubated with PrC‐210 (20 mmol L^−1^) added at various times (−1 hour to +1 minute) relative to addition of 5 mmol L^−1^ H_2_O_2_ and immediate irradiation of the tubes with UV light to catalyze essentially instantaneous conversion of H_2_O_2_ to •OH.^10^ Immediately following the 60‐second •OH pulse that is achieved during the 60‐second UV irradiation, triplicate samples of the irradiated plasmid DNA (260 ng) were electrophoresed on a 1% agarose gel in Tris‐acetate buffer for 90 minutes at 60 volts. Gels were stained with ethidium bromide, digitally imaged, and supercoiled vs nicked/•OH‐damaged DNA band intensities were quantified using Image J software.

### 2.9 Statistics

Data are expressed as means ± SEMs. One‐way Student *T* tests were used to determine statistical difference and *P* values using Graphpad Prism software.

## 3 RESULTS

### 3.1 PrC‐210 reduces ROS‐induced DNA damage to background

To compare both the *potency* and *efficacy* of PrC‐210 against “antioxidants” previously tested for suppression of myocardial ischemia‐reperfusion injury eg,^3^, ^7^, ^17^, ^21^, ^31^ we used an established gel‐based assay that uses naked plasmid DNA as a surrogate ROS target to score ROS‐induced damage, that is, plasmid DNA breaks (Figure 1A). This assay allowed direct molar comparison of ROS‐scavenging potency, and overall efficacy, between agents. PrC‐210 and WR‐1065 (the active metabolite of amifostine) were equally potent, and the other tested molecules virtually without effect in protecting the naked DNA against ROS (Figure 1B). At >10 mmol L^−1^ N‐acetylcysteine, a blood concentration that is probably achieved in clinical settings, N‐acetylcysteine actually increased ROS‐induced DNA damage. Glutathione was without discernible ROS‐scavenging effect; addition of the purified GSH‐transferase pi isoform enhanced DNA damage.

**Figure 2:**
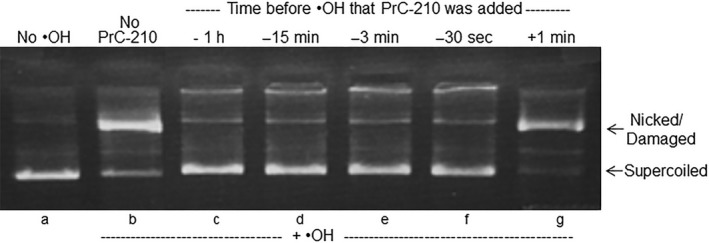
Agarose gel separation of supercoiled and nicked/•OH‐damaged forms of plasmid DNA after exposure of plasmid DNA to a 60 sec pulse of an •OH generator (H_2_O_2_ + UV light^10^. Supercoiled DNA was incubated with buffer (lane A) or buffer + 20 mmol L^−1^ PrC‐210 (lanes B‐G) for the indicated times and then exposed for 1 min to the •OH generator. Aliquots of each reaction were immediately electrophoresed, stained with EtBr and digitally imaged. Three replicate reactions and gels were done, and band intensities were quantified using Image J software

The *lead‐time* required for PrC‐210 to protect naked DNA by scavenging ROS was determined also using an assay of •OH‐induced plasmid DNA breaks (Figure 2). Here, a 60‐second pulse of •OH was generated, and PrC‐210 addition at every time point before the •OH insult, including as little as 30 seconds before, conferred complete protection against the •OH pulse that otherwise induced >95% damage to the naked DNA. PrC‐210 addition 1 minute after the ROS insult had no protective effect.

### 3.2 PrC‐210 reduces myocardial infarct size and troponin I

**Figure 3:**
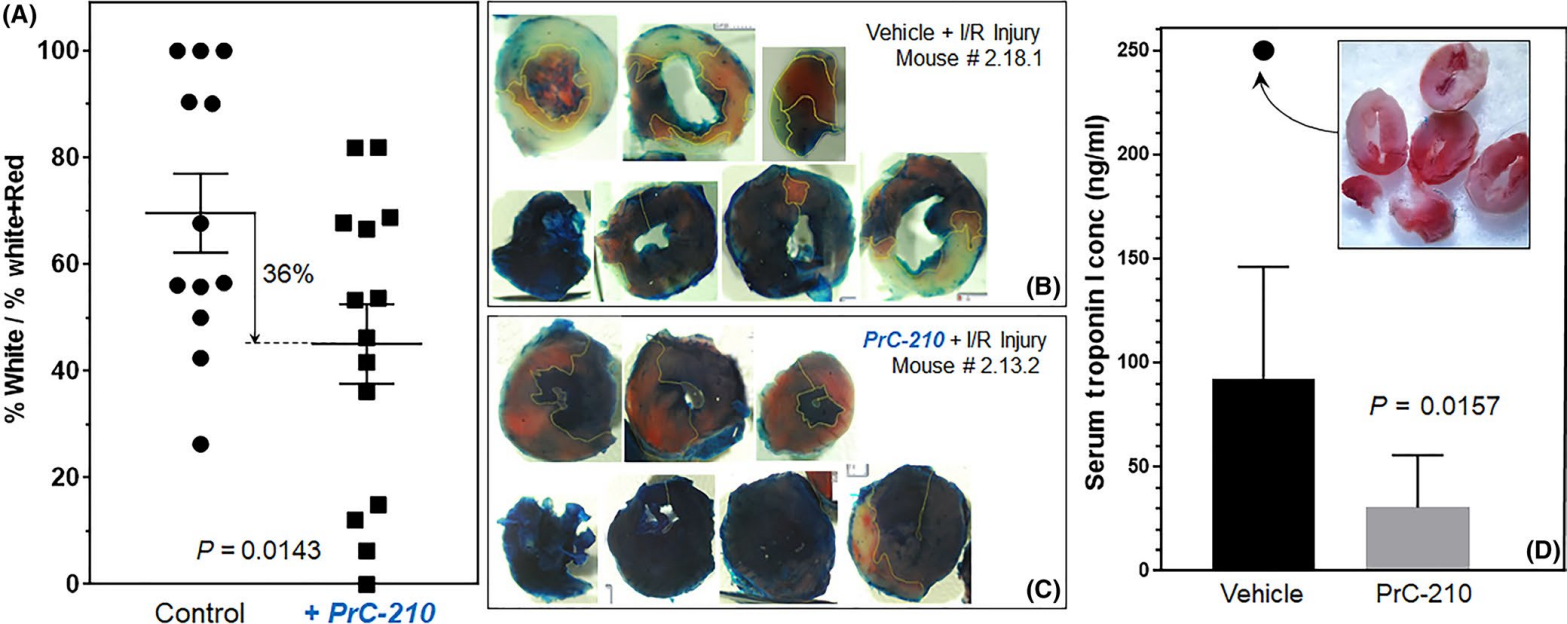
PrC‐210 reduced myocardial infarct size and serum Troponin I in ischemia/reperfusion mice. (A) Quantitative image analysis of red, white, and blue stained areas in sections of mouse hearts showed a significant, 36% reduction (*P* = 0.0143) in the white/white + red area percentage in mice that received an intraperitoneal PrC‐210 injection at the time of the 45‐min left coronary artery ligation. Each symbol shows the result from one mouse. Representative viability stained hearts from mice who received (B) vehicle or (C) PrC‐210 at the time of the 45‐min ischemia‐reperfusion injury. (D) Troponin I levels in mouse plasma 24 h after a 45 min cardiac ischemia‐reperfusion insult. The Troponin I level and unstained heart tissue sections from a control mouse whose coronary artery was ligated and not released for 24 h are also shown

With the above PrC‐210 efficacy in scavenging ROS, we asked whether PrC‐210 would be effective in scavenging ROS in the post‐ischemic, reperfused heart setting. To answer this question, PrC‐210 was tested using the standard mouse 45‐minute coronary artery ligation model. Each of the 26 mouse ischemia‐reperfusion hearts was TTC‐stained and sectioned. When compared to the vehicle control group, mice that received IP PrC‐210 had a significant reduction in the mean infarct size (36% reduction, *P* = 0.0143; Figure 3A‐C).

Plasma collected from IP vehicle and PrC‐210‐treated mice 24 hours after the cardiac ischemia‐reperfusion insult showed a significant reduction in the level of plasma Troponin I (67% reduction, *P* = 0.0157; Figure 3D).

### 3.3 PrC‐210 and functional performance of hearts following myocardial infarct

Cardiac function of post‐MI hearts was evaluated by echocardiography. The data show that there was a trend toward improved cardiac function in mice that received PrC‐210 (Table 1), but for most measured parameters the differences were not significantly different. Echocardiography imaging at 2 weeks and 4 weeks post‐MI showed that post‐MI hearts in PrC‐210 mice were on average 25% smaller in systole than MI hearts, and 16% smaller in diastole. Consistent with the smaller, less‐dilated hearts in PrC‐210 mice, (a) the ejection fractions in PrC‐210 mice were 19% greater, and (b) the ejection velocity through the aortic I valve in the PrC‐210 mice was 21% higher (*P* = 0.006) while heart rate was not different between treatments.

**Table 1:** In vivo metrics of cardiac function

Parameter		MI (N = 12)	MI + PrC‐210 (N = 9)	*P* value
Heart rate (bpm)		507	479	0.424
Ejection fraction (%)	+2 weeks	41.1	50.0	0.173
	+4 weeks	38.5	44.8	0.292
LV volume ‐ systole (μL)	+2 weeks	47.2	34.6	0.134
LV volume ‐ diastole (μL)		77.7	67.1	0.164
LV volume ‐ systole (μL)	+4 weeks	50.2	38.7	0.330
LV volume ‐ diastole (μL)		79.0	65.8	0.270
Ao VTI (mm/sec)		1333	1602	0.006

### 3.4 PrC‐210 reduces ROS‐induced death of neonate mouse cardiomyocytes

**Figure 4:**
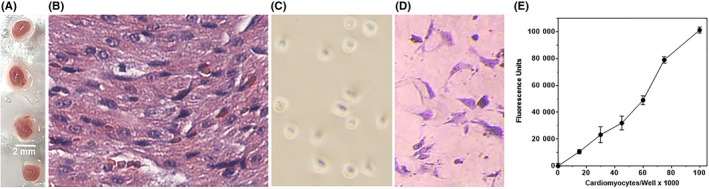
Isolation and primary culture of neonate mouse cardiomyocytes. (A) Neonate hearts (each heart weighed ~30 mg). (B) Hematoxylin and eosin stained histologic section of neonate mouse heart (100× magnification). (C) Cells from the collagenase digest of minced hearts that are attached to a noncoated Falcon tissue culture dish after a 60 min incubation in serum‐enriched medium; the unattached cells in the supernate from this dish were carried forward as cardiomyocytes. (D) Primary culture of neonate cardiac myocytes attached to a Matrigel‐coated 96 well plate; cells were stained with Giemsa. (E) Linear increase in neonate cardiomyocyte CellTiter fluorescence in Matrigel‐coated 96 wells seeded with the indicated numbers of neonate cardiomyocytes

To corroborate that PrC‐210 protected cardiac cells by directly scavenging ROS, neonate mouse cardiomyocyte cultures were directly exposed to H_2_O_2_ with or without PrC‐210 present. This model also allowed us to directly measure both the time‐ and dose‐dependency of PrC‐210 effect. Collagenase dispersion of neonate mouse hearts and enrichment of cardiomyocytes by panning out fibroblasts (Figure 4A‐C), provided primary cardiomyocyte cultures on Matrigel plates (Figure 4D), which linearly converted the CellTiter viability reagent in a cell number‐dependent manner (Figure 4E).

**Figure 5:**
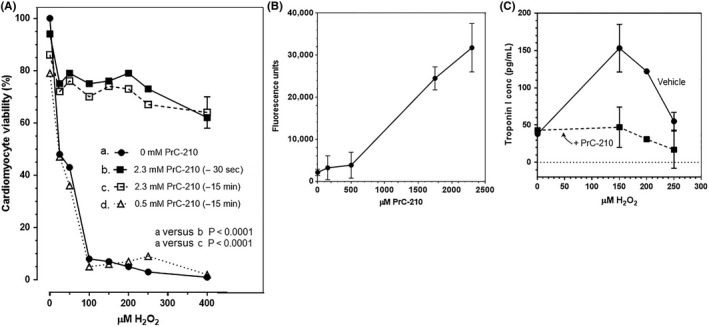
(A) Significant PrC‐210 reduction (*P* < 0.0001, a vs b or c) of H_2_O_2_‐induced neonate cardiomyocyte cell death. The shown error bar (SEM), which is ~10% of the mean value, is representative of the SEMs seen in all groups; error bars are not shown to better visualize curves. (B) PrC‐210 dose‐dependent protection of neonate cardiomyocytes that also received 200 µmol L^−1^ H_2_O_2_ in media 15 min after addition of PrC‐210 to media. Forty‐eight hr after cell seeding, CellTiter reagent was added, and after a 3‐h incubation at 37°C, fluorescence signal was measured. (C) PrC‐210 reduction of Troponin I release in H_2_O_2_‐treated primary cultures of neonate cardiac myocytes. Twenty‐four hr after seeding (75 000 cells/96 well), neonate myocytes were exposed to the indicated increasing concentrations of H_2_O_2_, either with or without 2.3 mmol L^−1^ PrC‐210 added to the media. After 2 h, 50 µL of media from each 96 well was transferred to a new plate, frozen at −80°C, and the media were subsequently thawed and assayed for Troponin I content by an ELISA assay

Addition of H_2_O_2_ to the cardiomyocyte cultures caused an H_2_O_2_‐dose‐dependent loss in cardiomyocyte viability, essentially to the zero‐cell background in the 200 µmol L^−1^ H_2_O_2_ wells (Figure 5A). Addition of 2.3 mmol L^−1^ PrC‐210 to the media in wells either 15 minutes or 30 seconds before H_2_O_2_ provided a profound increase in cardiomyocyte viability (80% in PrC‐210 + H_2_O_2_ vs 3% in H_2_O_2_ alone; *P* = 0.0001, Figure 5A). 2.3 mmol L^−1^ PrC‐210 was chosen because it is the highest blood plasma concentration of PrC‐210 thiol that was achieved^8^ after a mouse received an IP injection of the same 0.5 × MTD dose of PrC‐210 (ie, 252 µg/gm bw) that was used on the MI mice in this study. Addition of 0.5 mmol L^−1^ PrC‐210 to media 15 minutes before addition of H_2_O_2_, was without discernible protective effect (Figure 5A). Addition of increasing concentrations of PrC‐210, up to 2.3 mmol L^−1^, to cardiomyocyte wells 15 minutes before they received 200 µmol L^−1^ H_2_O_2_ showed a linear, PrC‐210 dose‐dependent increase in conferred protection (Figure 5B). Tissue culture media collected from cardiomyocyte wells showed a substantial reduction in the level of Troponin I (Figure 5C) in PrC‐210‐treated wells.

## 4 DISCUSSION

**Figure 6:**
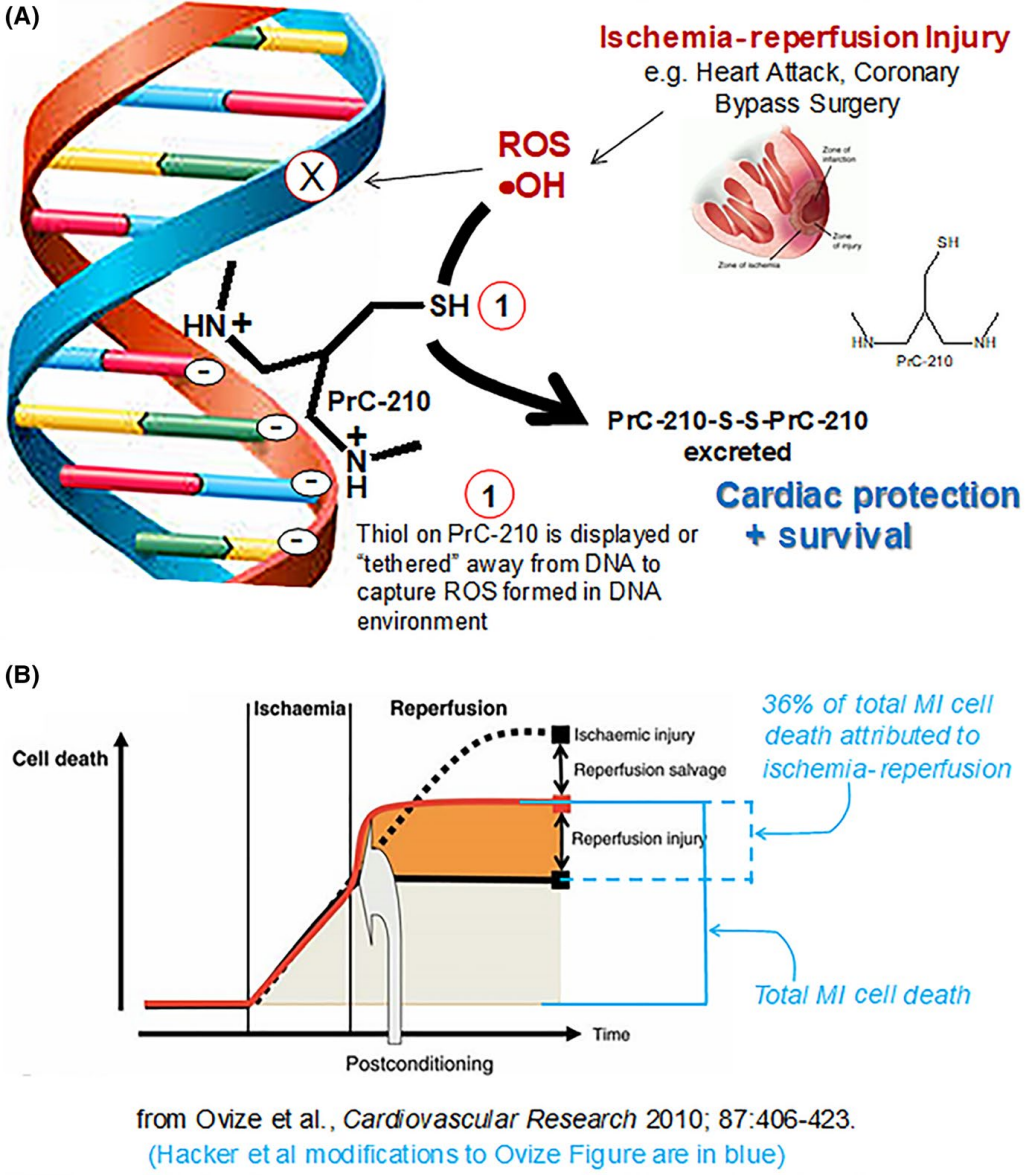
(A) Schematic representation of PrC‐210 mechanism of action via direct scavenging of ROS from the milieu immediately surrounding cellular DNA. (B) A schematic concept to explain cardiac ischemia‐reperfusion‐associated cell death during heart attack. Original image is from Ovize et al^20^; modifications to the original Ovize Figure are shown in blue

Ischemia‐reperfusion injury during heart attack remains a profound problem, and more broadly, IR‐injury also contributes considerable morbidity to many surgical subspecialties, including cardiac surgery. This study was done to determine if PrC‐210, an established ROS scavenger and radioprotectant, would be equally effective in preventing the ROS damage induced during the reperfusion that follows a heart attack. Our data demonstrate: (a) systemic PrC‐210 caused a significant 36% reduction of cardiac muscle death following a 45‐minute cardiac IR insult, (b) in a striking coincidence, the PrC‐210 36% reduction in cardiac muscle death equals the 36% of MI‐induced cardiac cell death estimated 6 years ago^20^ (Figure 6B) to result from “reperfusion injury,” (c) hearts in PrC‐210‐treated mice performed better after heart attacks when functionally analyzed using echocardiography, (4) the PrC‐210 ROS‐scavenging mechanism of action was corroborated by its ability to prevent 85% of the H_2_O_2_‐induced killing of neonate cardiomyocytes in cell culture, (5) PrC‐210 was the most potent and effective ROS‐scavenger when compared to eight of the commonly studied antioxidants in the heart attack literature, (6) PrC‐210 ROS‐scavenging efficacy is both immediate (seconds) and long‐lasting (hours), which makes it effective in both *a*)* real‐time *(*seconds*), as post‐MI or cardiac surgery hearts are reperfused with PrC‐210‐containing blood, and *b*)* long‐term *(*hours*)*,* as hearts are bathed with systemic PrC‐210 after MI or surgery, and (7) PrC‐210 does not cause the nausea, emesis, nor hypotension that preclude clinical use of the WR‐1065/amifostine aminothiol.^23^

A PubMed search of “cardiac ischemia‐reperfusion injury AND antioxidants” resulted in 7159 citations. These “antioxidant” reports primarily studied “antioxidants” (eg, Vitamin E) and nonantioxidant molecules (eg, adenosine) which have no direct effect upon ROS, and most commonly saw no suppression of IR injury eg.^3^, ^7^, ^17^, ^21^, ^31^ These earlier reports are important for having provided proof of concept for ROS‐suppression strategies, however, prolonged time to a protective effect and/or severe side effects of a known ROS scavenger (ie, amifostine/WR‐1065) prevented any of these molecules from becoming treatments in heart attack.

Traditional “antioxidants” have *zero* ROS‐scavenging capability, rather they are Michael acceptor activators of NrF‐2 and the downstream “protective” Phase 2 gene products activated by NrF‐2. This gene activation process requires hours to days to achieve protective effect, and the protection, *if any*, in a post‐MI heart arrives long after the cardiac reperfusion ROS bolus. Other published strategies have tested post‐MI inflammatory mediators, growth factors, and stem cells in an effort to decrease IR injury following cardiac ischemia‐reperfusion, for example.^19^ Although the above strategies may have some effect, they do not address the primary IR insult, which is the bolus of ROS that is generated within seconds to minutes following re‐establishment of blood flow to the previously infarcted heart tissue.

PrC‐210 is unlike *all of* the previously tested “antioxidants” in the heart attack literature. To our knowledge, PrC‐210 is currently the best available ROS‐scavenger. From the Figure 1 head‐to‐head comparison of PrC‐210 to previous “antioxidants” in the heart attack literature, we conclude: (a) PrC‐210 and WR‐1065 are equally potent and effective in suppressing ROS‐induced damage to naked DNA. However, PrC‐210 shows *zero* incidence of the retching/vomiting or hypotension toxicities that are induced by WR‐1065/amifostine and that preclude their clinical use^23^, ^26^ and (b) N‐acetylcysteine has no ROS‐scavenging capability, actually it enhanced X‐ray‐induced DNA damage at >10 mmol L^−1^, a concentration that may be achieved in a patient's blood in clinical settings. Rather, N‐acetylcysteine protection, when conferred, is better explained by its abilities to enhance synthesis of intracellular glutathione^15^ and induce expression of Phase 2 drug‐metabolizing genes.^28^

Several reviews^7^, ^20^ tie ROS‐induced damage to cardiac ischemia‐reperfusion injury. Ovize et al^20^ calculated the reperfusion injury portion of cardiac cell death in a heart attack, (a) which results from ROS, and (b) which could be prevented by a *direct‐acting ROS‐scavenger* if it was re‐perfused into the heart *“side‐by‐side” with the O_2._*

PrC‐210 could well be this *direct‐acting, ROS‐scavenger.*

Ovize and colleagues^20^ calculated that 36% of cardiac cell death was due to ROS‐induced “reperfusion injury” (see our Figure 6B). In a striking coincidence, in our first test of PrC‐210 effect against cardiac ischemia‐reperfusion, we observed a 36% suppression of cardiac cell death (see Figure 3A); that is the same percentage that Ovize estimated 6 years before.

PrC‐210’s *direct* mechanism of action as an ROS‐scavenger means it confers protection within seconds (Figure 2). Importantly, at time points ranging from 1 hour to 30 second before •OH insult, PrC‐210 prevented 100% of the ROS‐induced damage. DNA protection was not seen when PrC‐210 was added 1 minute after •OH exposure; therefore, PrC‐210 confers protection by directly scavenging short‐lived (milliseconds) ROS, *before* DNA damage occurs. These findings indicate that PrC‐210 has both rapid onset and a lasting effect when administered prior to the generation of ROS. This essentially “immediate” onset of action is a key characteristic that distinguishes PrC‐210 from all previously studied cardiac “antioxidants.”

Finally, the ability of PrC‐210 to confer 100% protection to DNA against ROS, regardless of when administered prior to ROS insult, combined with its small molecular weight (MW = 148), would allow PrC‐210 to be used in a variety of MI and cardiac surgery settings to reduce IR injury. For example, PrC‐210 could be: (a) administered IV to MI patients, both in ambulances (for “leakage” past cardiac blockages) and in the minutes before the angioplasty balloon is inflated so that PrC‐210 blood levels are “therapeutic” or even “super‐therapeutic” when the oxygenated blood re‐enters the ischemic cardiac muscle, (2) added to cardioplegia heart preservation solutions flushed through hearts before cardiac surgeries, (3) flushed directly into hearts immediately before implantation into heart transplant recipients, or (4) be given intravenously to a patient post‐MI or postopen heart surgery to suppress inflammatory cell‐induced ROS damage.

Importantly, the nature of PrC‐210 as a direct‐acting, highly effective ROS scavenger would allow it to be used in *any* environment in which blood flow is stopped and restarted (Figure 6A).

Ischemia‐reperfusion injury in heart attack remains a profound problem with significant implications for cardiac cell death and health care resource utilization in the MI aftermath. Therefore, it is imperative that improved strategies to prevent cardiac IR injury are developed. Here, we have shown PrC‐210 to be a highly effective ROS‐scavenger that significantly reduces IR injury‐associated cardiac cell death.
